# Perioperative coagulofibrinolytic responses in colorectal surgery patients without chemical thromboprophylaxis: a retrospective observational study

**DOI:** 10.1007/s00595-021-02393-4

**Published:** 2021-10-25

**Authors:** Hironori Matsumoto, Kei Ishimaru, Satoshi Kikuchi, Satoshi Akita, Yuji Yamamoto, Motohira Yoshida, Shigehiro Koga, Hiroyuki Egi, Yuji Watanabe

**Affiliations:** Division of Gastrointestinal Surgery and Surgical Oncology, Graduate School of Medicine, Ehime University, Shitsukawa, Toon, Ehime 791-0295 Japan

**Keywords:** Coagulofibrinolysis, Colorectal surgery, Venous thromboembolism

## Abstract

**Purpose:**

During the perioperative period, coagulofibrinolytic activation occurs, which occasionally results in thromboembolic complications. However, natural perioperative coagulofibrinolytic responses have not been well investigated. The present study examined perioperative coagulofibrinolytic changes and their association with the development of venous thromboembolism (VTE).

**Methods:**

We retrospectively analyzed the changes in coagulofibrinolytic markers for 7 days in 70 patients undergoing elective colorectal surgery. To explore the natural coagulofibrinolytic response, we investigated patients not undergoing perioperative chemical thromboprophylaxis.

**Results:**

Coagulation activation occurred from just after surgery to postoperative day (POD) 1, followed by a gradual decrease, but persisted to even POD 7. Fibrinolytic activity showed a tri-phasic response: activation, shutdown and reactivation. Consequently, fibrin/fibrinogen degradation product (FDP) and D-dimer levels continued to increase until POD 7. The development of deep vein thrombosis (DVT) was observed in 11 patients (15.7%). Postoperative sustained hyper-coagulation [soluble fibrin (SF) or thrombin–antithrombin complex (TAT) values on POD 7 > their normal limits] was significantly associated with the development of DVT (SF, *p* < 0.001; TAT, *p* = 0.001).

**Conclusion:**

We found initial coagulation activation and a tri-phasic response of fibrinolytic activity after colorectal surgery. Thus, physicians need to pay attention to these responses when attempting to prevent or treat VTE.

## Introduction

Physiological coagulation and fibrinolysis maintain hemostatic equilibrium under the delicate balance of bleeding and thrombus formation during the perioperative period [1]. Perioperative bleeding increases the risk of reoperation and the duration of hospital stay [2]. Thrombotic complications, such as deep vein thrombosis (DVT) and pulmonary embolism (PE), are infrequent but result in severe morbidity and mortality after surgery [3].

The coagulofibrinolytic status during the perioperative period changes in response to bleeding, thrombus formation and many other factors, including even surgical stress itself [4–6]. Coagulofibrinolytic markers are often used to evaluate the coagulofibrinolytic status, and previous reports have demonstrated that coagulation and fibrinolytic activation occur just after the initiation of surgery, which is then followed by a transient suppressed fibrinolytic phase (fibrinolytic shutdown) [4, 7, 8]. However, coagulofibrinolytic profiles during the postoperative phase may differ depending on surgical procedures, anesthesia and other factors [4–6]. Thus, it is necessary to evaluate the precise perioperative changes in coagulofibrinolytic markers according to different surgical techniques and conditions.

A high rate of venous thromboembolism (VTE) after colorectal surgery has been reported in some studies [9, 10]. Furthermore, while the development of VTE generally occurs in the early period after surgery, some studies have reported a high rate of development even after hospital discharge following colorectal surgery [9, 11, 12]. Therefore, the optimal method of perioperative chemical thromboprophylaxis needs to be discussed. Moreover, although coagulofibrinolytic markers, such as D-dimer, are considered useful for the early detection of VTE [13], their postsurgical accuracy is debatable, since perioperative coagulofibrinolytic changes lead to a high rate of false-positive results [14]. Thus, a systematic evaluation of perioperative coagulofibrinolytic changes in colorectal surgery, which carries a high risk of developing VTE, is needed.

Given the above, the present study examined the changes in coagulofibrinolytic markers during the perioperative period of colorectal surgery in patients with no chemical thromboprophylaxis, which will allow us to understand the changes in coagulofibrinolytic markers without any influence from pharmacological interventions.

## Methods

### Study design

We performed a retrospective observational study of patients who underwent elective colorectal surgery in Ehime University Hospital in Japan from September 2016 to December 2017. In accordance with our hospital policy during the study period, the patients were routinely examined for perioperative coagulofibrinolytic markers and DVT screening, and they did not generally receive chemical thromboprophylaxis. The patients were completely involved in the decision-making process, and written informed consent for the perioperative management was obtained from all patients.

This study complied with the Declaration of Helsinki and was approved by the Local Institutional Ethics Committee for Clinical Studies in Ehime University Hospital. The written informed consent requirement for this study was waived because of the retrospective design.

### Patients

We included patients undergoing elective colorectal surgery with no chemical thromboprophylaxis so that we could measure the actual changes in perioperative coagulofibrinolytic markers after the operation. “Elective colorectal surgery” in this case referred to any elective surgery with colorectal resection and primary anastomosis or diversion under general anesthesia. Patients were excluded if they had a clotting disorder, such as liver cirrhosis, had events of thromboembolic disease within three months before surgery, could not stop taking anticoagulants or antiplatelet agents for other diseases during the perioperative period or had received systemic chemotherapy within three weeks prior to surgery.

In general, the lithotomy position was used for laparoscopic surgery and the supine position for laparotomy. All patients had sequential compression devices placed on their lower extremities before surgery and continued postoperatively. Patients were encouraged to ambulate from postoperative day (POD) 1.

### Data collection and definition

The patients’ demographic characteristics were collected at the screening before surgery. Comorbidities included a history of heart diseases (including hypertension, angina, myocardial infarction and heart failure), respiratory diseases (including chronic obstructive pulmonary disease and bronchial asthma), chronic renal failure, diabetes, liver cirrhosis, neurological disorder (including stroke), steroid use and anticoagulant and/or antiplatelet agent use. Colorectal cancer was graded according to the Japanese Society for Cancer of the Colon and Rectum guidelines [15]. Post-operative complications were defined as Grade ≥ 2 for significant complications according to the Clavien–Dindo classification system [16]. DVT was evaluated preoperatively and on POD 7 by lower extremity duplex ultrasonography. The development of DVT was defined as a new blood clot or thrombus within the venous system.

Blood sampling was performed preoperatively, immediately after surgery and on PODs 1, 3 and 7. We measured routine blood counts and biochemistries with a TBA-c16000 (Toshiba Medical Systems, Tochigi, Japan) and XE-5000 (Sysmex, Hyogo, Japan). We also measured the biomarkers of coagulofibrinolysis using a CP-2000 (Sekisui Medical, Tokyo, Japan) and STACIA (LSI Medience, Tokyo, Japan). The biomarkers measured were as follows: prothrombin time (PT), activated partial thromboplastin time (APTT), fibrinogen (Fbg), fibrin/fibrinogen degradation product (FDP), D-dimer, thrombin–antithrombin complex (TAT), plasmin-α_2_-plasmin inhibitor complex (PIC), antithrombin (AT), protein C (PC), α_2_-plasmin inhibitor (α_2_PI) and plasminogen (PLG).

### Statistical analyses

Statistical analyses were performed using the IBM SPSS Statistics 22 software package (IBM, Tokyo, Japan). All data are expressed as the median with the interquartile range (IQR). Differences in patients’ clinical features, laboratory values and outcomes were assessed with Student’s *t *test, Mann–Whitney *U* test or Fisher’s exact test, as appropriate. Time course changes of values of coagulofibrinolytic markers during the study period were tested by a one-way repeated measures analysis of variance (ANOVA). Post hoc comparisons were made using the Bonferroni method or Mann–Whitney *U* test, as appropriate. A chi-square test was used for determining the association between the development of DVT and postoperative sustained hyper-coagulation. Postoperative sustained hyper-coagulation was diagnosed when the SF or TAT values on POD 7 were greater than or equal to their normal limits (SF, 7 μg/mL; TAT, 3 μg/L). We classified patients into binary categorical groups [high SF group: ≥ 7 μg/mL; and normal SF group: < 7 μg/mL; high TAT group: ≥ 3 μg/L; and normal TAT group: < 3 μg/L; DVT ( +) and DVT (−)]. A *p* value less than 0.05 was considered significant.

## Results

### Patient characteristics (Table 1)

**Table 1:** Patient characteristics and outcomes

	Total patients: *N* = 70	Development of VTE		
		VTE (+): *n* = 11	VTE (−): *n* = 59	*P* value
Sex (male/female), *n* (%)	41 (58.6)/29 (41.4)	5 (45.5)/6 (54.5)	36 (61.0)/23 (39.0)	0.263
Age, years	70 (64.8–77)	75 (67–81)	70 (63–76)	0.188
BMI, kg/m^2^	22.4 (20.3–24.3)	23.6 (21.0–25.2)	22.2 (20.2–24.3)	0.410
Smoking history, *n* (%)				0.422
Never	37 (52.9)	7 (63.6)	30 (50.8)	
Former	29 (41.4)	4 (36.4)	25 (42.4)	
Current	4 (5.7)	0 (0.0)	4 (6.8)	
Comorbidities, *n* (%)				
Heart diseases	19 (27.1)	2 (18.2)	17 (28.9)	0.375
Respiratory diseases	2 (2.9)	0 (0.0)	2 (3.4)	0.708
Chronic renal failure	0 (0.0)	0 (0.0)	0 (0.0)	*n.d*
Diabetes	12 (17.1)	3 (27.3)	9 (15.3)	0.281
Liver cirrhosis	0 (0.0)	0 (0.0)	0 (0.0)	n.d
Neurological disorders	1 (1.4)	0 (0.0)	1 (1.7)	0.843
Use of steroid	2 (2.9)	0 (0.0)	2 (3.4)	0.708
Use of anticoagulants and/or antiplatelet agents	17 (24.3)	2 (18.2)	15 (25.4)	0.467
Cause, *n* (%)				0.539
Cancer	66 (94.3)	10 (90.9)	56 (94.9)	
The Japanese society for cancer of the colon and rectum guidelines				
Stage I	16 (22.9)	3 (27.3)	13 (22.0)	
Stage II	26 (37.1)	3 (27.3)	23 (39.0)	
Stage III	17 (24.3)	4 (36.4)	13 (22.0)	
Stage IV	7 (10.0)	0 (0.0)	7 (11.9)	
Adenoma	1 (1.4)	0 (0.0)	1 (1.7)	
Mucocele of the appendix	2 (2.9)	1 (9.1)	1 (1.7)	
Malignant lymphoma	1 (1.4)	0 (0.0)	1 (1.7)	
Surgical approach (open/laparoscopic)	8 (11.4)/62 (88.6)	3 (27.2)/8 (72.7)	5 (8.5)/54 (91.5)	0.105
Surgical intervention, *n* (%)				0.070
Ileocecal resection	8 (11.4)	2 (18.2)	6 (10.2)	
Right hemicolectomy	12 (17.1)	1 (9.1)	11 (18.6)	
Transverse colectomy	1 (1.4)	0 (0.0)	1 (1.7)	
Left hemicolectomy	4 (5.7)	0 (0.0)	4 (6.8)	
Sigmoidectomy	11 (15.7)	2 (18.2)	9 (15.3)	
High anterior resection	4 (5.7)	0 (0.0)	4 (6.8)	
Low anterior resection	20 (28.6)	2 (18.2)	18 (30.5)	
Abdomino-perineal resection	6 (8.6)	3 (27.3)	3 (5.1)	
Hartmann operation	4 (5.7)	1 (9.1)	3 (5.1)	
Operation time, minutes	305.5 (210.8–371.3)	330.0 (169.0–439.0)	304.0 (212.0–366.0)	0.846
Amount of bleeding, mL	0 (0–105)	0 (0–535)	0 (0–100)	0.345
Transfusion, *n* (%)	3 (4.3)	1 (9.1)	2 (3.4)	0.406
RBC, U	0 (0–0)	0 (0–0)	0 (0–0)	
FFP, U	None	None	None	
PC, U	None	None	None	
Postoperative hospitalization, days	12 (7–199)	18 (10–34)	11 (10–16)	0.249
Outcome, *n* (%)				
In hospital mortality	0 (0.0)	0 (0.0)	0 (0.0)	*n.d*
Development of PE	0 (0.0)	0 (0.0)	0 (0.0)	*n.d*
Development of DVT	11 (15.7)			
Proximal/distal	0 (0.0)/11 (15.7)			
Symptomatic/asymptomatic	0 (0.0)/11 (15.7)			
Complications (Clavien–Dindo classification grade ≥ 2)	21 (30.0)	3 (27.3)	18 (30.5)	0.570
Paralytic ileus	9 (12.9)	1 (9.1)	8 (13.6)	0.568
Surgical-site infection	4 (5.7)	0 (0.0)	4 (6.8)	0.496
Anastomotic leakage	4 (5.7)	1 (9.1)	3 (5.1)	0.503
Anastomotic bleeding	2 (2.9)	0 (0.0)	2 (3.4)	0.708
Urinary tract infection	1 (1.4)	0 (0.0)	1 (1.7)	0.843
Bacterial translocation	1 (1.4)	1 (9.1)	0 (0.0)	0.157

Values are presented as the median (interquartile range) or number (%), if appropriate

BMI, body mass index; PRBC, packed red blood cells; FFP, fresh-frozen plasma; PC, platelet concentrates; VTE, venous thromboembolism; PE, pulmonary embolism; DVT, deep venous thrombosis; POD, postoperative day

Patient characteristics are shown in Table 1. Seventy patients (41 male, 29 female) with a median age of 70 (IQR: 64.8–77) years old and a median body mass index (BMI) of 22.4 (20.3–24.3) kg/m^2^ underwent elective colorectal surgery under general anesthesia. Nineteen patients (27.1%) had heart disease, including hypertension, and 17 patients used anticoagulant and/or antiplatelet agents. All patients included in this study were able to stop anticoagulant and antiplatelet agents during the perioperative period at the discretion of their cardiologists. The most common indication for surgery was cancer (*n* = 66, 94.3%), and 62 patients (88.6%) underwent laparoscopic surgery. The median operation time was 305.5 (210.8–371.3) minutes, and the median amount of bleeding was 0 (0–105) mL. The median postoperative hospitalization duration was 12 (7–199) days.

Three patients (4.3%) had asymptomatic DVT preoperatively, and postoperative development of DVT was observed in 11 patients (15.7%). All DVT cases were asymptomatic and of the distal type. Symptomatic PE did not occur in any patients. No patients developed symptomatic PE or DVT during the 30-day postsurgical observation period. Other complications occurred in 21 patients (30.0%), including paralytic ileus (*n* = 9, 12.9%), surgical-site infection (*n* = 4, 5.7%), anastomotic leakage (*n* = 4, 5.7%) and anastomotic bleeding (*n* = 2, 2.9%). A comparison of the characteristics of patients with VTE and without VTE is shown in Table 1. The two groups were similar.

### Perioperative changes in coagulofibrinolytic markers (Table 2 and Fig. 1)

**Table 2:** Preoperative laboratory data

Preoperative laboratory data (*N* = 70) [normal range]		
WBC [3500–9000]	5400 (4800–6725)	/μL
HGB [11.3–15.2]	12.1 (10.3–14.1)	g/dL
HCT [35.0–52.0]	36.9 (32.5–42.0)	%
PLT [15.0–40.0]	22.4 (17.2–30.5)	× 10^4^/μL
SF [< 7.0]	3.0 (3.0–3.0)	μg/mL
TAT [< 3.0]	1.2 (0.9–1.6)	μg/L
APTT [21.5–43.1]	26.7 (25.4–29.4)	Sec
PT [80.0–120.0]	95.8 (86.7–112.0)	%
HPT [70.0–130.0]	101.8 (92.5–123.5)	%
Fbg [200–400]	333.5 (298.3–403.8)	mg/dL
AT [80.0–120.0]	95.5 (85.9–108.2)	%
PC [82.0–112.0]	97.3 (86.0–109.4)	%
PLG [80.0–130.0]	100.4 (90.6–112.3)	%
α_2_PI [80.0–130.0]	102.1 (91.7–109.7)	%
FDP [< 5.0]	3.2 (2.5–4.5)	μg/mL
D-dimer [< 1.0]	0.9 (0.6–1.3)	μg/mL
PIC [0.0–0.8]	1.0 (0.8–1.5)	μg/mL
CRP [< 0.3]	0.1 (0.1–0.4)	mg/dL

Values are presented as the median (25–75th percentiles)

**Fig. 1:**
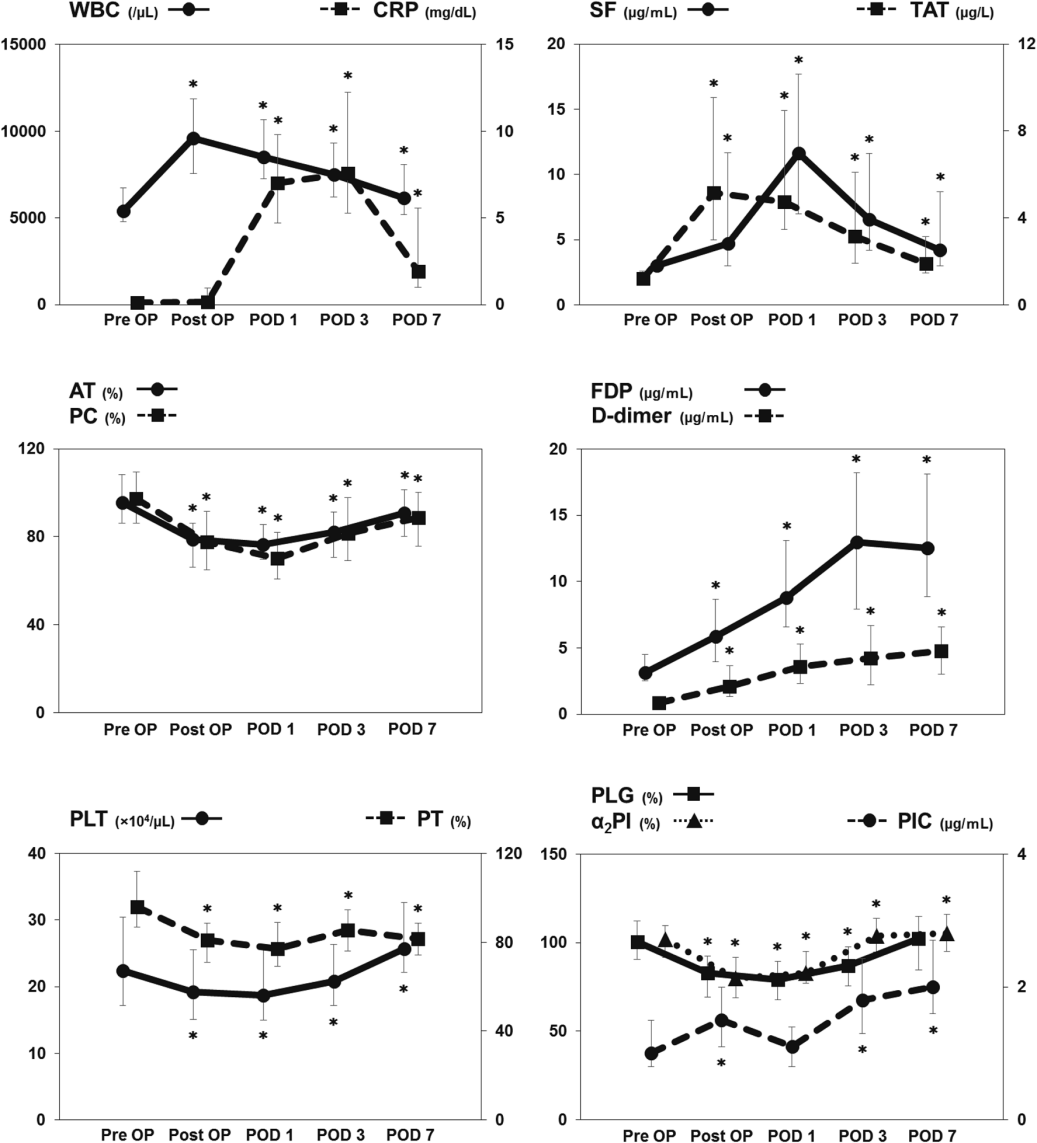
Perioperative time course changes in coagulofibrinolytic markers. Lines represent the median values, and the upper and lower whiskers represent the 25–75th percentiles, respectively. *p < 0.05 comparison between the postoperative values and the preoperative values

The median values of preoperative laboratory data are shown in Table 2. Time course changes in perioperative coagulofibrinolytic markers are shown in Fig. 1. WBC significantly increased after surgery as compared with the preoperative values (*p* < 0.0001), followed by a gradual decrease. CRP also significantly increased from POD 1 to 3 (*p* < 0.0001) and then gradually decreased. Among the parameters of coagulation activity, SF and TAT significantly increased after surgery [SF on POD 1, 11.7 μg/mL (4.7–6.05), *p* < 0.0001; TAT postoperatively, 5.2 μg/L (2.15–4.38), *p* < 0.0001]. These values decreased gradually to their normal ranges but remained higher than before the operation until POD 7 [SF, 4.2 μg/mL (3.0–8.7), *p* < 0.0001; TAT, 1.9 μg/L (1.5–3.1), *p* = 0.022]. Regarding the fibrinolytic activity, PIC significantly increased immediately after surgery [1.5 μg/mL )1.1–2.0), *p* < 0.0001] and then returned to the preoperative value on POD 1 [1.1 μg/mL (0.8–1.4), *p* = 0.985]. Subsequently, PIC increased again and stayed elevated until POD 7 [2.0 μg/mL (1.6–2.7), *p* < 0.0001]. FDP and D-dimer remained at increased levels throughout the postoperative period [FDP on POD 7, 12.5 μg/mL (8.9–18.1), *p* < 0.0001; D-dimer on POD 7, 4.8 μg/mL (3.0–6.6), *p* < 0.0001]. PLT and PT were slightly decreased after the operation compared to the preoperative values [PLT on POD 1, 19.3 × 10^4^/μL (15.1–25.6), *p* < 0.0001; PT on POD 1, 81.0% (71.0–88.5%), *p* < 0.0001]. AT and PC, markers of the anticoagulation state, were also slightly but significantly decreased after surgery, followed by a gradual recovery [AT on POD 2, 76.4% (69.7–85.6%), *p* < 0.0001; PC on POD 2, 70.1% (60.5–82.0%), *p* < 0.0001]. PLG and α_2_PI also showed a slight decrease from the baseline values after surgery [PLG on POD 2, 79.0% (67.6–89.2%), *p* < 0.0001; α_2_PI on POD 1, 79.8% (68.9–91.5%), *p* < 0.0001]. Even though they slowly returned to normal ranges, the PT, AT and PC levels on POD 7 remained lower than the preoperative baseline [PT, 81.6% (74.3–88.6%), *p* < 0.0001; AT, 90.6% (80.0–101.3%), *p* < 0.0001; PC%, 88.6% (75.5–100.1%), *p* < 0.0001].

### Correlation between sustained postoperative sustained hypercoagulation and development of DVT (Table 3)

**Table 3:** Correlations between the postoperative hyper-coagulation and the perioperative development of DVT

	*χ* ^*2*^	df	*p* value	OR	95% CI
SF	10.329	1	0.001	8.571	1.998–36.767
TAT	16.687	1	< 0.001	17.625	3.357–95.523

df, degree of freedom; OR, odds ratio; CI, confidence interval

Regarding SF and TAT on POD 7, 31.4% (22/70) and 27.1% (19/70) of all patients, respectively, showed values higher than normal (SF, 7 μg/mL; TAT, 3 μg/L). The High-SF and High-TAT groups were defined as having postoperative sustained hyper-coagulation. The chi-square test revealed a remarkable correlation between the perioperative development of DVT with postoperative sustained hyper-coagulation [SF, *χ*^*2*^ = 10.329, degree of freedom (df) = 1, *p* = 0.001, odds ratio (OR): 8.571, 95% confidence interval (CI): 1.998–36.767; TAT, *χ*^*2*^ = 16.687, df = 1, *p* < 0.001, OR: 17.625, 95%CI: 3.357–92.523].

## Discussion

The present study examined the natural perioperative coagulofibrinolytic responses to surgical stress. We evaluated the perioperative changes in coagulofibrinolytic markers in colorectal surgery patients who did not receive perioperative chemical thromboprophylaxis. We measured the levels of TAT and SF as indicators of coagulation activity and PIC as an indicator of a fibrinolytic state. Antithrombin binds intravascular thrombin in a 1:1 ratio to inhibit thrombin effects, and in doing so, it forms TAT; therefore, TAT reflects intravascular thrombin formation. Soluble fibrin is generated from thrombin–fibrinogen reactions, making it a marker of intravascular coagulation as well. Activated plasmin lyses fibrinogen and fibrin to generate FDP and D-dimer and is then inactivated immediately by α_2_PI to form PIC. Thus, PIC reflects fibrinolytic activity.

**Fig. 2:**
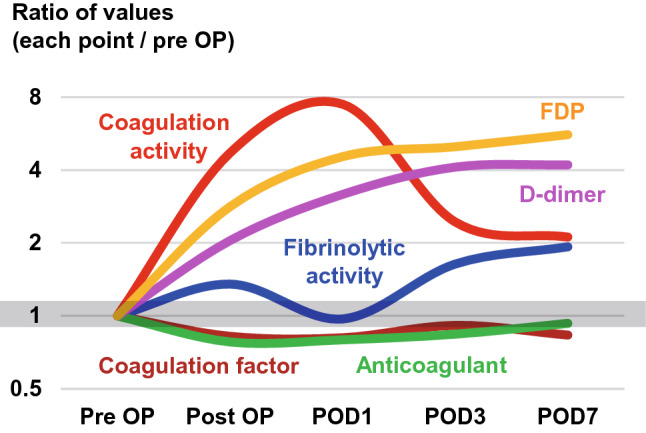
Chronological changes in the perioperative coagulofibrinolytic status. The vertical axis shows the ratio of postoperative values to the preoperative values. Coagulation activity, TAT; fibrinolytic activity, PIC; coagulation factor, PT activity; anticoagulant, AT

As be expected, coagulation activation was observed immediately after surgery until POD 1, and then there was a gradual decline in coagulation activation. In contrast, fibrinolytic activity showed a tri-phasic response: activation, then shutdown, followed by reactivation. FDP and D-dimer levels continued to increase until POD 7. A schematic diagram of the chronological changes in the coagulofibrinolytic profile after surgery is shown in Fig. 2.

### Coagulofibrinolytic responses in colorectal surgery

Initial coagulation activation and the tri-phasic fibrinolytic response observed in the current study have also been reported in previous studies involving other types of surgery or trauma [4, 7, 8]. Immediately after surgical or traumatic insult, coagulation and fibrinolytic activation occur simultaneously. Fibrinolytic inhibition due to increasing levels of plasminogen activator inhibitor-1 (PAI-1), a controller of excessive fibrinolysis, then gradually develops after the initial coagulofibrinolytic activation. Upon the repair of injured vessels and tissues, PAI-1 levels decrease to reactivate fibrinolysis for the removal of the fibrin attached to the vessels to induce hemostasis. However, these coagulofibrinolytic responses might differ depending on the degree or duration of trauma or surgical procedures [4–6]. The responses seen in the current study were actually similar but much smaller than those observed in our previous study of trauma patients [17]. Furthermore, a previous report demonstrated a significant difference in fibrinolytic activity between orthopedic and abdominal surgeries [4]. Thus, it was necessary to evaluate the surgeries performed without pharmacological interference to separately evaluate the perioperative changes in the coagulofibrinolytic markers of colorectal surgery. In this respect, this may be the first report to demonstrate the coagulofibrinolytic responses in colorectal surgery.

Furthermore, since we studied patients who did not receive perioperative chemical thromboprophylaxis, which can affect coagulofibrinolytic responses, our results might reflect actual physiological responses to the surgical stress. In such study conditions, we found that considerable levels of coagulation and fibrinolytic activation occurred, and FDP and D-dimer levels continued to increase until POD 7. However, we should bear in mind that the perioperative coagulofibrinolytic responses of our results may have been partly affected by postoperative factors, such as development of VTE and complications, which were observed in general. These results are likely to demonstrate the natural perioperative course, including such general postoperative factors, and we must understand the basic changes.

### Sustained hyper-coagulation and development of DVT

A high incidence of VTE after colorectal surgery, a major abdominal surgery, has been reported in previous studies [9, 10], so perioperative chemical thromboprophylaxis after high-risk major abdominal surgery is recommended by the American College of Chest Physicians Evidence-Based Clinical Practice Guidelines [18]. While the development of VTE usually occurs in the early postoperative period, some studies have indicated a high occurrence rate of VTE even after hospital discharge in colorectal surgery [9, 11, 12]. Therefore, the ideal schedule and dosage of anticoagulants for perioperative chemical thromboprophylaxis remains controversial [12, 19–21].

In the current study, we found that sustained hyper-coagulation was evident even at POD 7 in approximately 30% of all patients, and DVT occurred in 15.7%. Furthermore, our analyses demonstrated that sustained hyper-coagulation was significantly associated with the development of DVT during the hospital stay. This finding is consistent with previous reports that found that the postoperative hyper-coagulation state plays a key role in the development of VTE [22, 23]. In addition, early postoperative fibrinolytic shutdown, which was also found in the present study, might have contributed to thrombus formation [24, 25]. The VTE cases in the present study were all asymptomatic and distal DVT, which might be less prone to embolize into the pulmonary circulation than symptomatic or proximal DVT. However, a new onset of such thrombus should be considered a significant clinical thrombotic tendency. We may thus reasonably conclude that patients in a prolonged hyper-coagulation state might have a potential risk of developing VTE during their hospital stay and possibly even after discharge. Thus, we need to consider anticoagulant therapy, especially for patients who show postoperative sustained hyper-coagulation. We also need to identify it early as possible and determine the risk factors for such patients.

### The perioperative evaluation of coagulofibrinolytic markers for VTE

While several types of screening, such as monitoring D-dimer levels, are extensively used in the clinical settings to assess the development of VTE, the coagulofibrinolytic markers TAT and SF have been reported to be useful for assessing the risk for developing VTE [26]. In particular, D-dimer screening is internationally recognized as a highly accurate test for detecting VTE [13]. However, its efficacy after surgery is not always optimal. Since perioperative coagulofibrinolytic marker levels, including D-dimer, might be affected by surgical stress, these changes can lead to a high rate of false-positive results in the diagnosis of VTE [14, 23]. Indeed, the positive predictive cut-off values of D-dimer for VTE in previous studies have varied widely by surgery type [27–30]. One reason for the high rate of false-positive results might be a postoperative increase in D-dimer levels [31]. Indeed, in the current study, the FDP and D-dimer levels increased soon after surgery and continued to increase until POD 7. Postoperative FDP and D-dimer increases may be due to physiological fibrinolytic activation after the initial coagulation activation. Thus, when we evaluate coagulofibrinolytic markers for predicting VTE development, we need to consider the various factors affecting the perioperative coagulofibrinolytic responses, such as the sequential physiological changes after the surgery itself, surgical method and postoperative complications, including the development of VTE. Based on the understanding of the natural perioperative coagulofibrinolytic responses demonstrated in our results, further examinations of the influence of many factors on the perioperative coagulofibrinolytic responses are needed to clarify the appropriate cut-off point for detecting VTE.

### Limitations

Several limitations associated with the present study should be addressed. First, the sample size of this study was small and drawn from a single center. Second, we excluded patients who could not discontinue perioperative anticoagulant therapy due to exacerbation of preexisting thromboembolism. Despite the fact that the patients included in this study seemed to have a relatively low risk of VTE, we aimed to determine the natural coagulofibrinolytic responses to surgery itself. Third, the sustained hyper-coagulation as described in this study may have been due to physiological responses to the surgery itself, so such hyper-coagulation is not always useful for detecting every instance of perioperative DVT. We examined the development of DVT in the early postoperative period, but a high rate of DVT can occur even after hospital discharge in colorectal surgery, so a further study will be needed to investigate the later coagulofibrinolytic changes after colorectal surgery. Fourth, in addition to surgery, there are many factors associated with coagulofibrinolytic markers or the development of VTE, such as the stage of cancer or general anesthesia. In the future, large-scale studies will be needed to test the hypotheses that have arisen from the present study.

## Conclusion

Coagulofibrinolytic markers considerably changed during the perioperative period, and these changes persisted beyond seven days after colorectal surgery. We need to consider these responses when deciding on prophylaxis or assessing the risk of perioperative VTE.

## Data Availability

The datasets for the current study are available from the corresponding author upon reasonable request.

## Abbreviations

AT: Antithrombin
TAT: Thrombin–antithrombin complex
Alb: Albumin
PC: Protein C
PT: Prothrombin time
APTT: Activated partial thromboplastin time
Fbg: Fibrinogen
FDP: Fibrin/fibrinogen degradation product
PIC: Plasmin-α_2_-plasmin inhibitor complex
α_2_PI: α_2_-Plasmin inhibitor
PLG: Plasminogen
